# Monitoring the Neurotransmitter Response to Glycemic Changes Using an Advanced Magnetic Resonance Spectroscopy Protocol at 7T

**DOI:** 10.3389/fneur.2021.698675

**Published:** 2021-08-18

**Authors:** Young Woo Park, Dinesh K. Deelchand, James M. Joers, Anjali Kumar, Alison Bunio Alvear, Amir Moheet, Elizabeth R. Seaquist, Gülin Öz

**Affiliations:** ^1^Department of Radiology, Center for Magnetic Resonance Research, University of Minnesota, Minneapolis, MN, United States; ^2^Department of Medicine, University of Minnesota, Minneapolis, MN, United States

**Keywords:** magnetic resonance spectography, ultrahighfield MRI, dielectric pad, prospective motion correction, brain metabolism

## Abstract

The primary excitatory and inhibitory neurotransmitters glutamate (Glu) and gamma-aminobutyric acid (GABA) are thought to be involved in the response of the brain to changes in glycemia. Therefore, their reliable measurement is critical for understanding the dynamics of these responses. The concentrations of Glu and GABA, as well as glucose (Glc) in brain tissue, can be measured *in vivo* using proton (^1^H) magnetic resonance spectroscopy (MRS). Advanced MRS methodology at ultrahigh field allows reliable monitoring of these metabolites under changing metabolic states. However, the long acquisition times needed for these experiments while maintaining blood Glc levels at predetermined targets present many challenges. We present an advanced MRS acquisition protocol that combines commercial 7T hardware (Siemens Scanner and Nova Medical head coil), BaTiO_3_ dielectric padding, optical motion tracking, and dynamic frequency and B_0_ shim updates to ensure the acquisition of reproducibly high-quality data. Data were acquired with a semi-LASER sequence [repetition time/echo time (TR/TE) = 5,000/26 ms] from volumes of interest (VOIs) in the prefrontal cortex (PFC) and hypothalamus (HTL). Five healthy volunteers were scanned to evaluate the effect of the BaTiO_3_ pads on ${\text{B}}_{1}^{+}$ distribution. Use of BaTiO_3_ padding resulted in a 60% gain in signal-to-noise ratio in the PFC VOI over the acquisition without the pad. The protocol was tested in six patients with type 1 diabetes during a clamp study where euglycemic (~100 mg/dL) and hypoglycemic (~50 mg/dL) blood Glc levels were maintained in the scanner. The new protocol allowed retention of all HTL data compared with our prior experience of having to exclude approximately half of the HTL data in similar clamp experiments in the 7T scanner due to subject motion. The advanced MRS protocol showed excellent data quality (reliable quantification of 11–12 metabolites) and stability (*p* > 0.05 for both signal-to-noise ratio and water linewidths) between euglycemia and hypoglycemia. Decreased brain Glc levels under hypoglycemia were reliably detected in both VOIs. In addition, mean Glu level trended lower at hypoglycemia than euglycemia for both VOIs, consistent with prior observations in the occipital cortex. This protocol will allow robust mechanistic investigations of the primary neurotransmitters, Glu and GABA, under changing glycemic conditions.

## Introduction

Glutamate (Glu) and gamma-aminobutyric acid (GABA) are primary excitatory and inhibitory neurotransmitters, respectively, in the central nervous system. Alterations to Glu and GABA concentrations have been linked to various neurological and psychiatric disorders (1–4). The metabolism of Glu and GABA is also closely linked to oxidative glucose (Glc) utilization (5), as both Glu (6) and GABA (7) are involved in the metabolic pathways of the brain. Studies with animal (8, 9) and human subjects (10, 11) have indicated that the Glu and GABA levels decrease as the brain is exposed to hypoglycemia. In addition, clinical studies on impaired awareness of hypoglycemia (IAH) in patients with type 1 diabetes (T1D) have shown differences in the Glu response to hypoglycemia in patients with vs. without IAH, suggesting underlying changes in the cognitive mechanisms of hypoglycemia awareness (10, 12, 13).

Hence, understanding how the concentrations of Glu and GABA change in response to changing glycemia can provide important insights into cerebral adaptations in diabetes and recurrent hypoglycemia (14). While the metabolic pathways that couple glycemia to neurotransmitter levels are active across brain regions, the hypothalamus (HTL) and the prefrontal cortex (PFC) are notable regions of interest (ROIs) to understand these adaptations (15). Namely, the HTL contains the Glc-sensing neurons and controls the release of counter-regulatory hormones such as glucagon, epinephrine, growth hormone, and cortisol to maintain peripheral Glc homeostasis (16, 17). The PFC is the primary brain region controlling executive function, and differences in Glc kinetics in this region have been associated with impaired hypoglycemia awareness (18, 19).

Magnetic resonance spectroscopy (MRS) is an MR modality that provides access to brain metabolism and allows direct *in vivo* quantification of endogenous, high-concentration metabolites. Therefore, ^1^H MRS allows simultaneous measurement of a neurochemical profile, including Glu and GABA, and Glc. Use of ultrahigh-field (UHF) MRI allows reliable detection of metabolites with complex spectral pattern such as Glu, GABA, glutathione (GSH), and lactate (Lac), as the higher field yields enhanced signal-to-noise ratio (SNR) and allows greater separation of spectral peaks. Earlier metabolic MRI and MRS studies at UHF have primarily investigated the brain's metabolic responses to glycemic changes in the occipital cortex (OCC), using surface coils that were needed to achieve the required radio frequency (RF) transmit energy (${\text{B}}_{1}^{+}$) levels but lacked whole-brain coverage (10, 20). Access to other parts of the brain requires the use of volume coils and ideally commercially available equipment to enable investigations by a community of researchers. While MRS using X-nuclei, such as ^2^H and ^13^C, provides direct access to real-time measurements of metabolism, the need for administering labeled precursors and non-standard hardware provides motivation to develop and utilize ^1^H MRS methods to gain insights into metabolism.

Many prior studies have acquired MRS data in conjunction with the Glc clamp technique to observe how the body reacts to changing glycemic conditions (10–13, 21, 22). Glc clamp is a technique to maintain the plasma Glc at targeted levels by carefully controlling the amount of dextrose and insulin given to the subject. Measuring the brain's response to changing glycemic conditions with MRS requires a carefully choreographed coordination in and around the subject with frequent blood draws during the scan. These studies often require long acquisition times, typically around 2–3 h for 2 volumes of interest (VOIs), to enable adequate monitoring of physiological changes, which make the experiments prone to subject motion and decreases reliability of the MRS signal. In addition, streamlining the setup and calibration processes for MR data acquisition is critical for maximizing comfort of study participants and minimizing disruptions for clinical staff.

Therefore, our goal in this study was to develop a robust MRS protocol to minimize data loss and provide high sensitivity to neurotransmitter changes. We developed a UHF MRS data acquisition protocol with prospective motion and shim corrections to enable reliable measurements of neurotransmitter levels during glycemic clamps in the scanner. The proposed advanced MRS acquisition protocol combines commercial 7T hardware (7T Siemens scanner and Nova Medical head coil) and BaTiO_3_ dielectric padding to streamline the scanner setup while achieving necessary ${\text{B}}_{1}^{+}$ levels for short-echo ^1^H MRS with an optimized semi-LASER (sLASER) protocol. In addition, the sLASER sequence incorporates optical motion tracking, and dynamic frequency and B_0_ shim updates to ensure the acquisition of reproducibly high-quality data during the long Glc clamp sessions. The optimized protocol was evaluated in the PFC and HTL, regions associated with IAH, in patients with T1D during consecutive euglycemic and hypoglycemic clamps.

## Methods

### MR Protocol

#### MR Hardware and Dielectric Padding

MR experiments were performed using a 7 tesla (T) Actively Shielded MAGNETOM scanner (Syngo VB17A, Siemens Healthineers, Erlangen, Germany) with proton (^1^H) single channel RF transmit (Tx) and 32-channel RF receive (Rx) head coil (Nova Medical, Wilmington, MA, USA). The Nova coil shares the same design as the Nova 1Tx/32Rx head coil for Siemens 7T Terra scanner, which is the one of two Food and Drug Administration (FDA)-approved commercial UHF system as of date. Due to well-known RF inhomogeneities at 7T, acquiring high-quality MRS data from the outer cortical regions of the brain is challenging. The transmit RF power is focused at the center of the coil and leads to insufficient power delivery to the cortical regions within the specific absorption rate (SAR) limits set for the human brain.

In order to overcome these challenges, we utilized dielectric pads to redistribute RF energy from the center of the brain toward our ROI, the PFC, by placing the pad on the forehead. Dielectric pads were prepared from a class of titanium oxide-based compounds (ceramic powders) that possess favorable dielectric properties. Specifically, water mixtures of barium titanate (BaTiO_3_, CAS#12047-27-7) were used as described previously (23, 24). BaTiO_3_ is harmful if swallowed, owing to the toxicity of the free Ba^2+^ that is liberated in acidic conditions, such as one's stomach (25). To mitigate formation of Ba^2+^ within the pads and preserve stability of BaTiO_3_, a mixture of deionized heavy water (D_2_O, 99.8% D atom) and Darvan (deflocculant) was titrated to pH = 9 with the addition of sodium hydroxide (NaOH) prior to the addition of the powdered BaTiO_3_. During the creation of the pads, a double heat seal was created along the pad edge, and the pad was double bagged to further prevent the direct skin exposure to BaTiO_3_.

#### Advanced Magnetic Resonance Spectroscopy Protocol With Motion Correction

The semi-LASER pulse sequence [sLASER, repetition time/echo time (TR/TE) = 5,000/26 ms] (26) was used to minimize chemical-shift displacement errors. GOIA-WURST adiabatic pulse was used, as it offered optimal pulse profile for refocusing (27). VAPOR water suppression (28) combined with outer volume suppression was used prior to sLASER localization.

**Figure 1:**
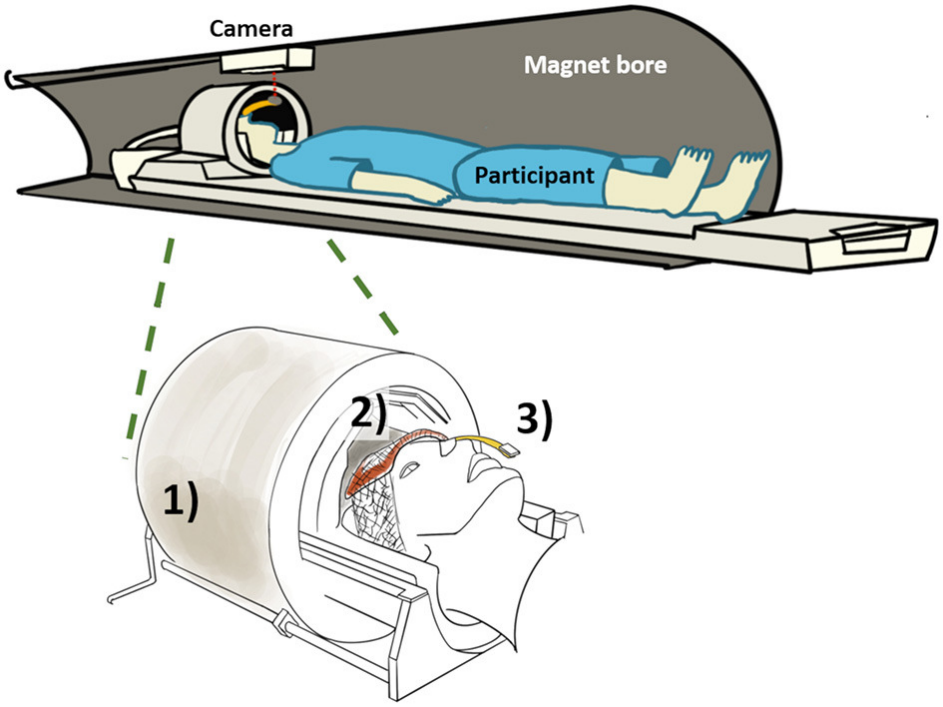
Overview of proposed method with (1) commercial head coil, (2) BaTiO_3_ padding on forehead, and (3) holographic optical marker attached to a plastic extension bar and taped to the participant's nose bridge.

In order to ensure consistent data quality throughout the long study, prospective motion correction was implemented, which performed real-time VOI position updates and corresponding corrections to first-order B_0_ shim values and incorporated a frequency navigator for efficient water suppression (29). An optical motion tracking system (MPT high field; Metria Innovation, Inc., Milwaukee, WI, USA) was used to compensate for head movements (Figure 1). The MR-compatible single optical camera (Metria Innovation, Inc.) installed at the isocenter in the bore of MRI scanner reads the position and orientation of the Moiré Phase Tracking (MPT) marker attached to the participant's head. The MPT marker was attached to a plastic extender that was taped to the nose bridge as the transmit component of the Nova coil obstructs the MPT marker if directly placed on the nose. The camera system records the position of MPT in the camera coordinates that are translated to scanner coordinates using the XPACE library (KinetiCor, Inc., Calgary, AB, Canada). The head position is recorded at the beginning of the session, and XPACE library automatically computes and adjusts the position during the scan for prospective motion corrections.

First- and second-order B0 shimming was achieved with FASTMAP shimming prior to the start of MRS data acquisition (30). During the MRS acquisition, the first-order shim value was dynamically updated for each TR. A 2D RF pulse (duration of 6.51 ms) with small flip angle (20°) was transmitted just prior to the sLASER pulse sequence, and the information collected (three echoes with TE = 2.6 ms and an echo delay of 5.5 ms) was used to compute the first-order linear shim values (total duration of B_0_ shimming module at 85.53 ms) (29). In addition, two frequency navigator pulses (slice navigator pulse with 5° flip angle and STEAM navigator pulse with 10° flip angle) were transmitted before and after the dynamic shim module to compensate for shift in resonance frequency. The advantage of using low fractional anisotropy (FA) 2D RF pulse compared with the conventional FASTMAP (with five adiabatic pulses) is the significantly lower power deposition, which is beneficial at high fields. In addition, the delay between the acquisitions of B_0_ projections can be minimal without any SAR issues (0.02% contribution to total energy deposited).

### Protocol Evaluation

#### Participant Recruitment and Screening

The study protocol was approved by the Institutional Review Board (IRB) at the University of Minnesota. For the evaluation of dielectric padding, healthy participants were screened and consented on the day of the scan. The inclusion criterion were adult (18 years or older) healthy volunteers, and the exclusion criterion was inability to undergo MRI scanning (presence of paramagnetic substances or pacemakers in body, weight over 300 lb, pregnancy, and claustrophobia). For the evaluation with Glc clamps, patients with T1D were initially screened and consented 1 week prior to the scan. The inclusion criteria were ages 18–65 years with disease duration of 2–30 years and A1C <8.5%. The exclusion criteria were impaired awareness to hypoglycemia as determined by Cox (31) and Gold (32) questionnaire, uncontrolled hypertension with blood pressure >145/95 mmHg, evidence of autonomic neuropathy, proliferative retinopathy, impaired kidney function with glomerular filtration rate <45, history of cardiac diseases, current substance abuse, and inability to undergo MRI scanning. Clinical information (disease duration and A1C) was collected at the screening visit. On the date of the study, the subjects underwent the consent and MR safety screening again.

#### Effect of the Dielectric Padding

Effect of the BaTiO_3_ padding on ${\text{B}}_{1}^{+}$ and SNR was evaluated in five healthy volunteers (two females, mean ± SD age 43 ± 21 years). Each participant underwent imaging and MRS with and without the BaTiO_3_ pad. The pad was placed on the subject's forehead using Surgilast (Derma Sciences, Plainsboro, NJ, USA) tubular elastic dressing retainer. MRS data were acquired from the PFC (24 × 12 × 30 mm^3^, 16–64 transients) and HTL (13 × 12 × 10 mm^3^, 32 transients). ${\text{B}}_{1}^{+}$ efficiency was measured with actual flip-angle imaging (AFI) pulse sequence (33) at Tx 190–220 V.

#### Data Acquisition With Proposed Protocol During Glucose Clamps

The MRS protocol was evaluated in six patients (four females) with T1D. The age of the participants was 29 ± 9 years with the duration of the disease at 19 ± 5 years and well-maintained A1C level of 7.7 ± 0.6. Nova 32Ch head coil with BaTiO_3_ padding was used for the data acquisition. Semi-LASER pulse sequence (TR/TE = 5,000/26 ms) with motion tracking was used for the acquisition of MRS data.

**Figure 2:**
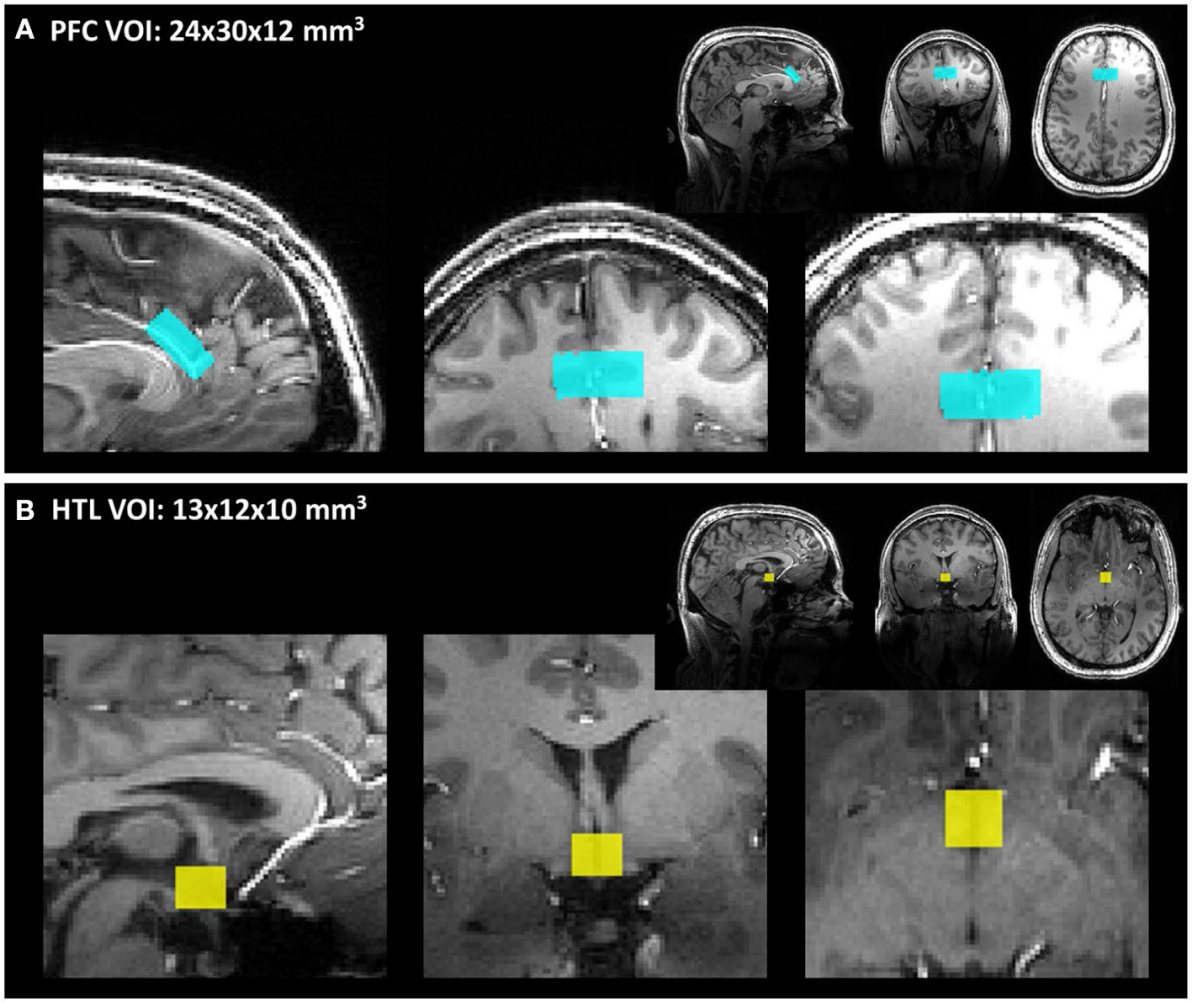
**(A)** Prefrontal cortex (PFC) and **(B)** hypothalamus (HTL) volumes of interests (VOIs) shown on T_1_-weighted magnetization-prepared rapid acquisition gradient echo (MPRAGE) images.

**Figure 3:**
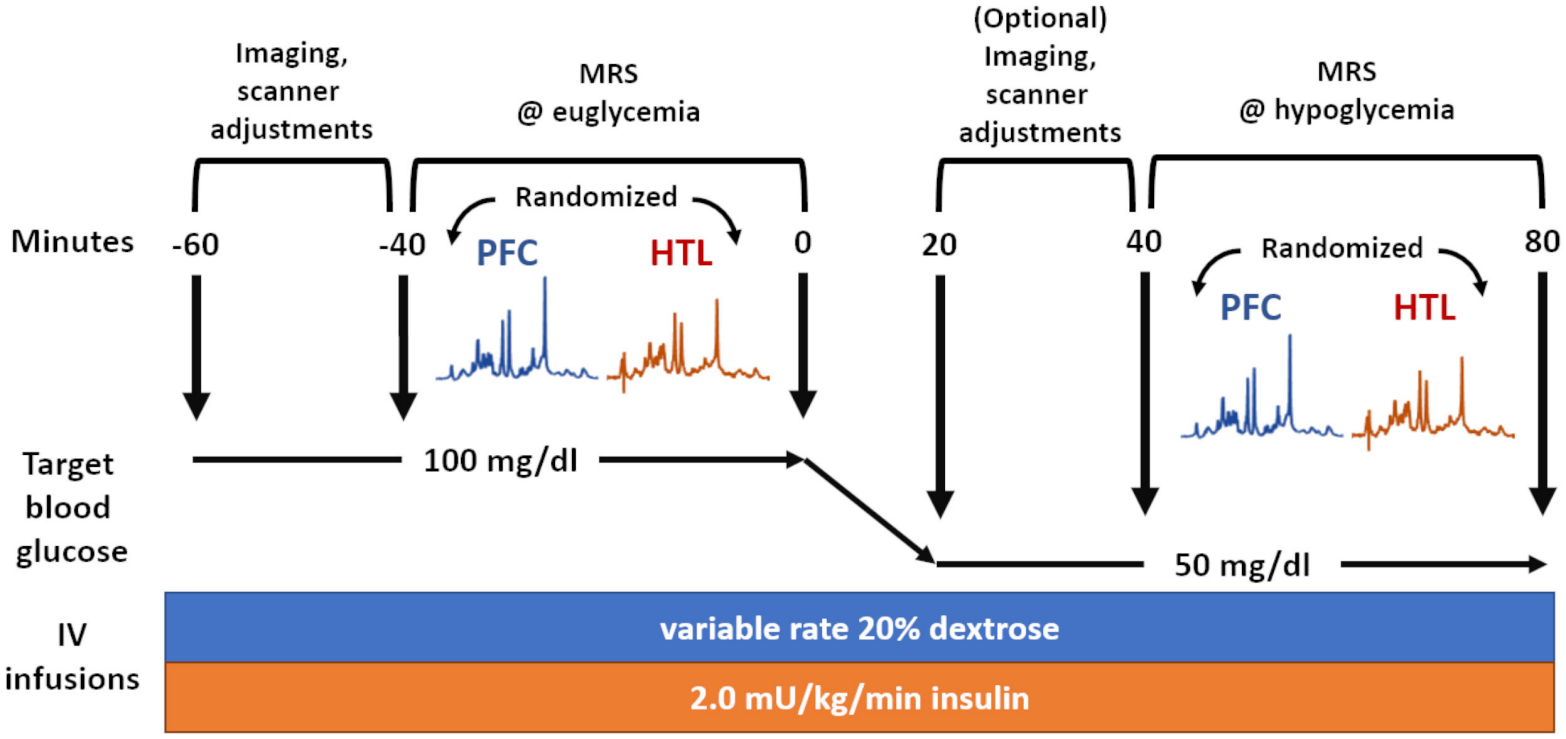
Overview of magnetic resonance spectroscopy (MRS) data acquisition during glucose clamps where the same set of data is acquired during euglycemia and hypoglycemia.

VOIs for the MRS data were the PFC (24 × 12 × 30 mm^3^, 64 transients) and HTL (13 × 12 × 10 mm^3^, 256 transients) (Figure 2). The order of MRS measurements in the PFC and HTL was randomized between participants but stayed the same between euglycemia and hypoglycemia (Figure 3). Total time of acquisition was about 6 min for the PFC and 22 min for the HTL with 3–5 min of voxel-based B_0_ and ${\text{B}}_{1}^{+}$ calibrations preceding each acquisition. Two additional sets of water unsuppressed sLASER spectra (two transients each) were collected for eddy-current correction and metabolite quantification (34).

Additional imaging data were acquired prior to MRS acquisitions. Structural images for MRS VOI localization and tissue segmentations were first collected with T_1_-weighted MPRAGE sequence at 1 × 1 × 1 mm^3^ resolution and FA 5° with TR/inversion time (TI)/TE = 2,890/1,500/2.42 ms. Additional proton-density (PD)-weighted FLASH images at 1 × 1 × 1 mm^3^ resolution and FA 5° with TR/TE = 1,410/2.42 ms were collected to normalize intensity for tissue segmentation. ${\text{B}}_{1}^{+}$ map with AFI was acquired between T_1_ and PD images (3 × 3 × 4 mm^3^ resolution, TR/TE = 60/2.33 ms, 60° flip angle @ Tx = 220 V), in order to compute the Tx power for MRS VOI.

An intravenous (IV) catheter was placed antegrade in a forearm of the subject for a two-step hyperinsulinemic clamp, in which insulin at a rate of 2.0 mU/kg/min and potassium phosphate at a rate of 4 mEq/h were infused. In addition, separate IV catheters were placed retrograde in one or both lower feet (as tolerated) for blood sampling. We set up IV lines in both feet when tolerated, as the lines sometimes failed to draw blood after a prolonged study session. Blood was sampled primarily on one line and only moved to another line if the initial line failed. The foot used for blood sampling was wrapped in heated towels to arterialize the venous blood (35). Blood sampling occurred every 5 min from the moment the IV lines became active before the MR scan. This was uninterrupted during the MR operation unless the subject's IV lines failed or the subject had to take a restroom break. Subjects were scanned while undergoing euglycemic and hypoglycemic Glc clamps. Blood Glc was initially maintained at euglycemia (100 mg/dL) by IV infusion of 20% dextrose. After collection of MR data during euglycemia, the dextrose infusion was temporarily halted, and blood Glc was allowed to drop to hypoglycemia (50 mg/dL). Once the plasma Glc had reached the target concentration, dextrose infusion was adjusted to maintain targeted hypoglycemia, while the same set of MR data was acquired (Figure 3). Heart rate was monitored using a pulse oximeter.

**Figure 4:**
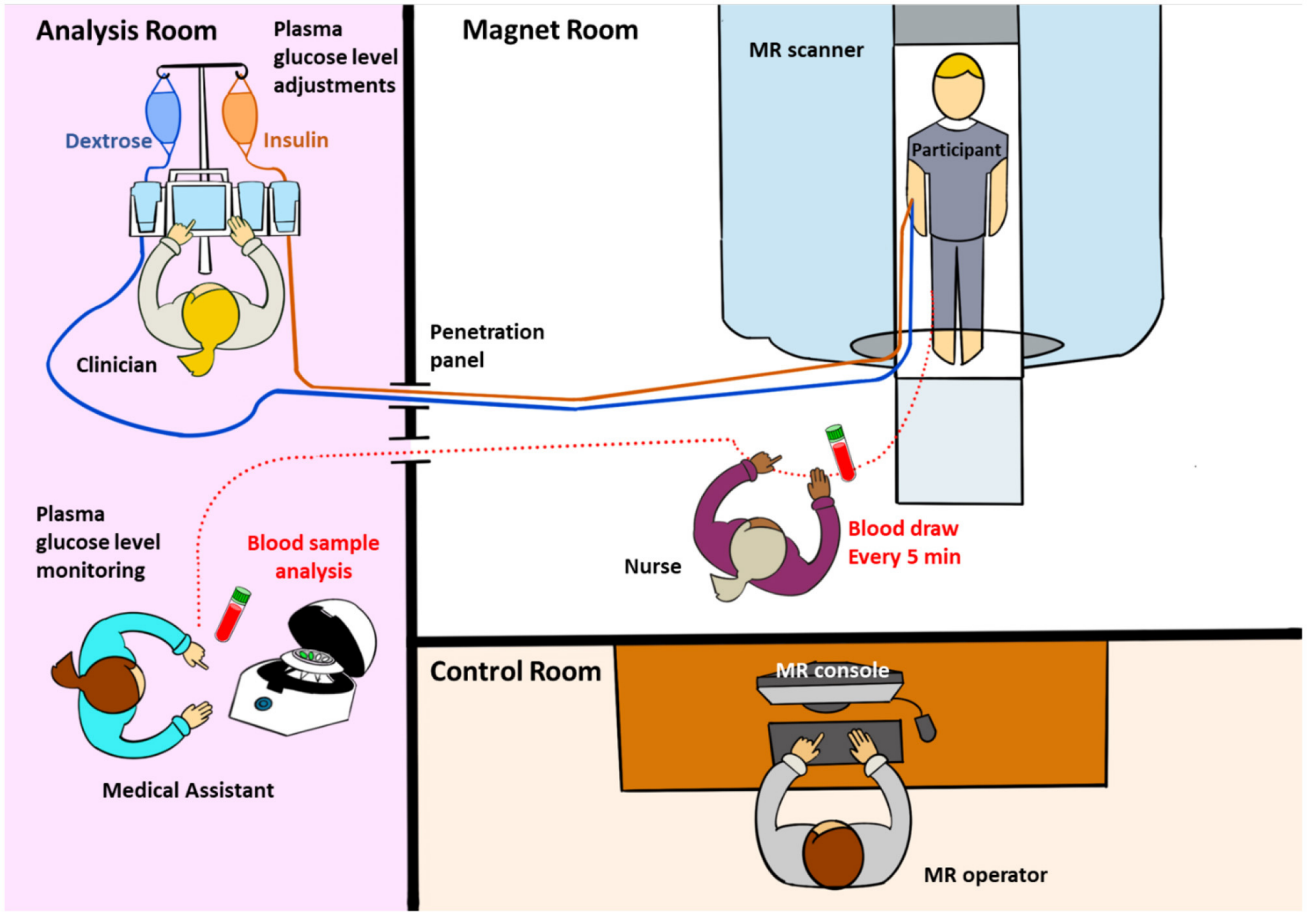
MRI/magnetic resonance spectroscopy (MRS) data acquisition during glucose clamp.

The MRI and MRS data acquisition during Glc clamp required coordination between three clinical staff and the MR operator (Figure 4). Blood Glc was sampled every 5 min to monitor Glc concentration, resulting in one to two samples for each 6-min-long MRS acquisition for the PFC, and four to five samples for each 22-min-long MRS acquisitions for the HTL. With continuous IV fluid injections, five out of six subjects needed to take a bathroom break after the euglycemic clamp. In those cases, T_1_-weighted structural images and AFI images for ${\text{B}}_{1}^{+}$ calibrations were repeated for VOI repositioning and sLASER RF power calibrations prior to data acquisition during hypoglycemia. The total MR session took about 2.5–3 h to complete.

#### Post-processing of Data

Channel-combined single-shot MRS spectra were saved to a DICOM file on the scanner. MATLAB-based MRspa software (36) was used to perform eddy-current, frequency, and phase corrections of single-shot spectra before averaging. Averaged MR spectra were quantified using LCModel (6.3-0G) with a density-matrix simulated basis dataset, as described previously (37). The simulated basis set includes aspartate (Asp), glutamine (Gln), myo-inositol (Ins), phosphoethanolamine (PE), phosphocholine (PCho), glycerylphosphorylcholine (GPC), creatine (Cr), phosphocreatine (PCr), *N*-acetylaspartate (NAA), *N*-acetylaspartylglutamate (NAAG), and taurine (Tau), in addition to GABA, Glu, GSH, Glc, and Lac.

Metabolite concentrations were corrected for T_2_ relaxation of tissue water and cerebrospinal fluid (CSF) content. CSF fraction within each VOI was estimated using image segmentation. A Matlab (v2019b, MathWorks, Natick, MA, USA) script was used to generate voxel masks over T_1_ and PD structural images. Tissue segmentation was performed in SPM12 using T_1_ and PD structural images. Tissue water fraction values were set to 84% for the PFC and 75.8% for the HTL (38). As the apparent T_2_ relaxation rate is slower due to the Carr–Purcell (CP) conditions of semi-LASER, we used corrected T_2_ relaxation times of water to estimate metabolite concentrations under CP conditions. We assumed that the T_2_ of water under CP conditions is 1.5× longer than the measured free precession T_2_ based on a previous study that compared water T_2_ values measured with LASER and CP-LASER sequences (34, 39). Hence, T_2_ of tissue water was set to 87 ms for the PFC (37) and 66 ms for the HTL after the measured free precession T_2_ values for primarily gray- and white-matter VOI from prior work (40) were multiplied by 1.5. Metabolites with between-subject mean Cramér–Rao lower bounds (CRLB) equal to or <20% were reported. Sum of metabolites were reported if two metabolites showed strong negative correlation (*r* < −0.7), or if one of the individual concentrations in Glu+Gln or Glc+Tau did not meet the mean CRLB ≤20% criterion.

#### Spectral Quality Metrics and Statistical Analysis

SNR and linewidth of the water reference spectra were used as quantitative measures for spectral quality. SNR was measured by dividing the amplitude (in the frequency domain) of the NAA peak by the root mean squared error (RMSE) of the spectral baseline between −5 and 0 ppm in averaged, non-weighted spectra. As the euglycemia and hypoglycemia datasets were collected from the same subject, we performed two-tailed, paired *t*-tests on the mean blood Glc, SNR, linewidth, and LCModel quantification results to evaluate differences between euglycemia and hypoglycemia for each VOI. Multiple comparison correction was not performed. *p*-Values of 0.05 or lower were considered significant.

## Results

### Effects of Dielectric Padding

**Figure 5:**
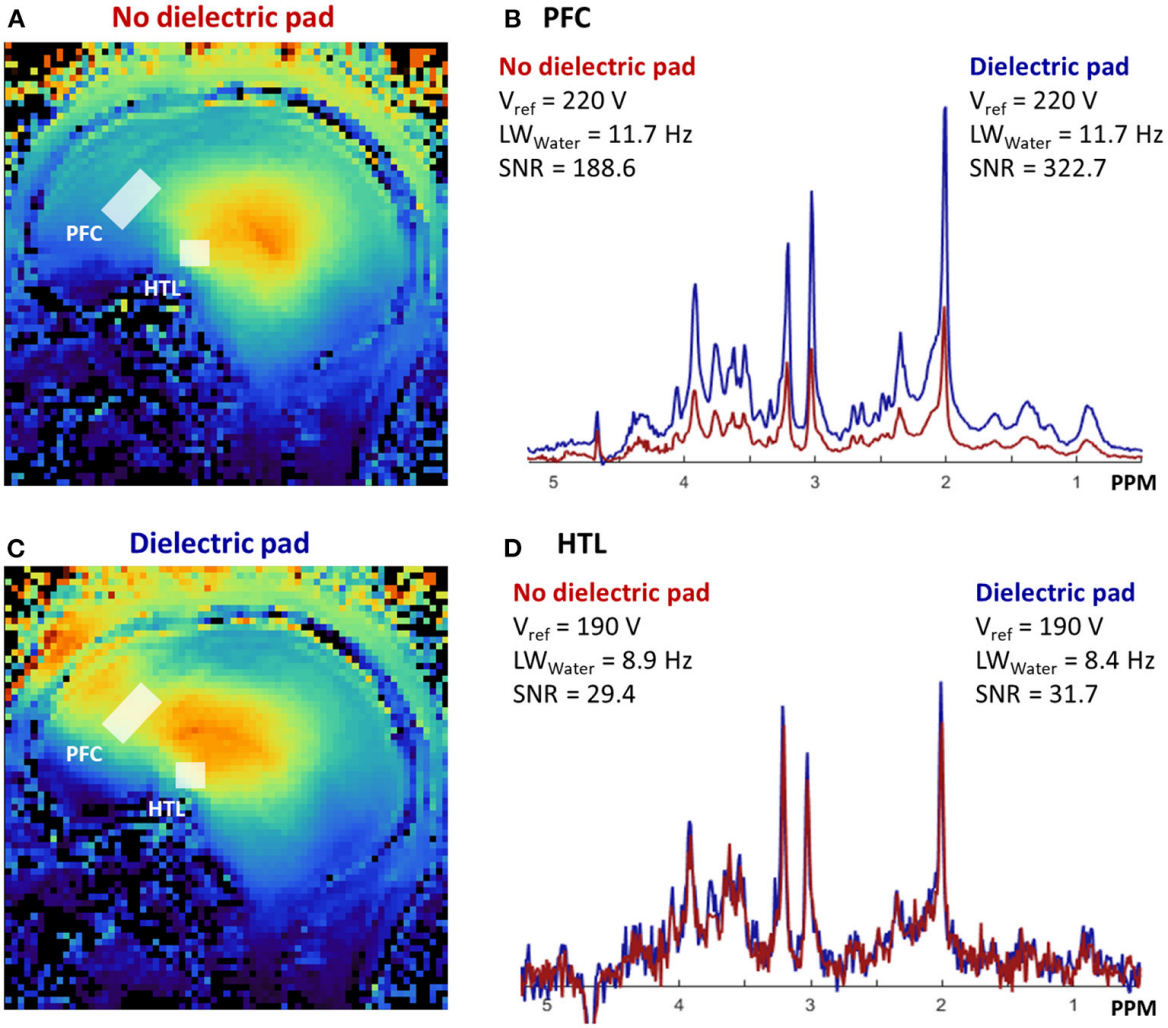
${\text{B}}_{1}^{+}$ map comparisons **(A,C)** show improvement of ${\text{B}}_{1}^{+}$ in the frontal cortex (PFC) with BaTiO_3_ pad placed on the subject's forehead. **(B)** About 60% gain in signal-to-noise ratio (SNR) was achieved in sLASER MR spectra (TE/TR = 5,000/26 ms) in PFC (64 transients) with the pad. **(D)** Smaller SNR gain was seen in hypothalamus (HTL) (32 transients), because the ${\text{B}}_{1}^{+}$ distribution of the coil (without padding) is the highest in the center of the brain, close to the HTL location.

Dielectric pads produced with BaTiO_3_ water mixtures allowed production of pads with high dielectric constants while having a thin profile of 5.5 mm. Scans with and without the BaTiO_3_ pad showed that ${\text{B}}_{1}^{+}$ in the frontal cortex was substantially improved by use of the pad (Figure 5). Simulation of the sLASER pulse sequence showed that the RF power necessary for 90° excitation is 26.1 μT and that the GOIA-WURST refocusing pulses in sLASER would lose adiabaticity if the RF power is below 18.8 μT. Actual RF power delivered to the PFC was 16.5–17.8 μT without dielectric pad and 21.3–28.2 μT with the pad. For the HTL, the values were 23.5–29.1 μT without pad and 23.9–35.6 μT with the pad. Consistently, the improvement in signal quality was more profound in the PFC (54–82% gain in SNR) than in the HTL (8–18% gain in SNR) when using the pad (Figures 5B,D).

### Data Quality During Glucose Clamps

MR data were acquired during Glc clamps using a BaTiO_3_ pad with dimensions of 180 × 100 × 5.5 mm^3^. While few acquisitions reached the SAR limit, most stayed under the limit. We have limited the Tx voltage to 235 V as a safety precaution, which in some subjects was well-below the SAR limit imposed by the system. Mean ${\text{B}}_{1}^{+}$ to the PFC was 23.7 ± 1.1 μT and to the HTL was 24.6 ± 1.9 μT (*N* = 6).

**Figure 6:**
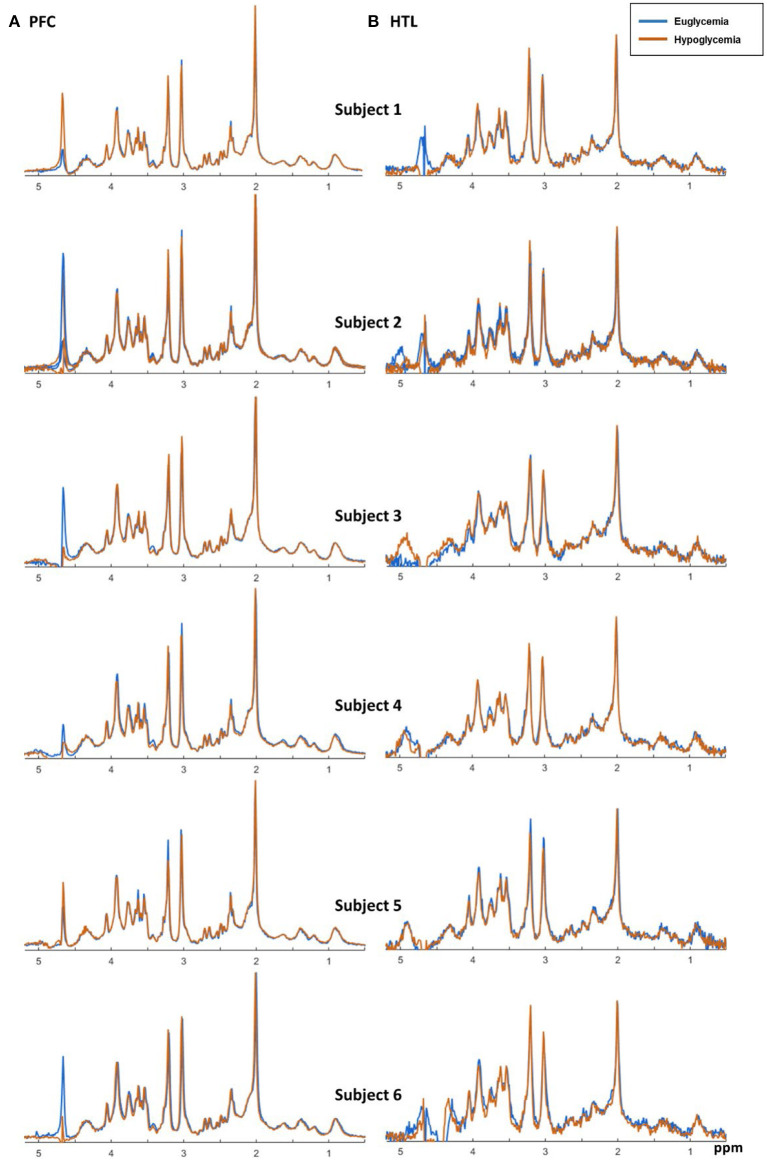
Semi-LASER MR spectra (7T, TR/TE = 5,000/26 ms, 64 transients for frontal cortex (PFC) **(A)** and 256 transients for hypothalamus (HTL) **(B)**) acquired from six subjects during glucose clamps.

Real-time motion, frequency, and first-order B0 shim correction allowed preservation of spectral quality during euglycemic and hypoglycemic Glc clamps. The spectra acquired from both regions had good quality with flat baseline, excellent SNR, and no unwanted coherences. Spectral quality was consistent between euglycemia and hypoglycemia (Figure 6).

Across-subject means of plasma Glc showed that glycemia was well-maintained in the target range during data acquisition from each VOI (Table 1). Spectral quality metrics of PFC and HTL spectra obtained during euglycemia and hypoglycemia did not show any significant differences (Table 2). All water reference linewidths were well-below the recommended 19-Hz threshold for excluding spectra (41); therefore, no spectra were excluded from analysis. In addition, no single shots were excluded due to poor quality for PFC datasets (64 transients). For the HTL, all but one subject (*N* = 5) had complete data (256 transients) for the analysis. In the one subject with incomplete HTL data, two single shots were removed from the euglycemia dataset, and one shot was removed from the hypoglycemia dataset.

**Table 1:** Mean plasma glucose values for both volumes of interests (VOIs) during euglycemic and hypoglycemic glucose clamps in patients with type 1 diabetes (*N* = 6).

**Plasma glucose** **(mg/dL)**	**PFC**			**HTL**		
	**Eu**	**Hypo**	***p*-Value**	**Eu**	**Hypo**	***p*-Value**
Mean (SD)	108.6 (9.8)	48.9 (3.5)	*p* < 0.0001	104.6 (9.8)	50.2 (6.1)	*p* < 0.0001

*Two-tailed two-sample unequal variance t-test was performed to compare plasma glucose levels between euglycemia and hypoglycemia*.

*PFC, prefrontal cortex; HTL, hypothalamus*.

**Table 2:** Signal-to-noise ratio (SNR) and linewidth (full-width at half-maximum) of water reference spectra for both volumes of interests (VOIs) during euglycemic and hypoglycemic glucose clamps in patients with type 1 diabetes (*N* = 6).

**Mean (SD)**	**VOI**	**Euglycemia**	**Hypoglycemia**	***p*-value**
SNR	PFC	366.3 (67.5)	364.9 (64.1)	0.97
	HTL	70.8 (10.5)	68.8 (6.2)	0.60
Linewidth in Hz	PFC	10.9 (1.1)	10.8 (0.5)	0.83
	HTL	11.5 (3.1)	10.6 (2.6)	0.66

*Paired, two-tailed t-tests show no significant differences in SNR and linewidths between euglycemia and hypoglycemia*.

*SNR, signal-to-noise ratio; PFC, prefrontal cortex; HTL, hypothalamus*.

**Figure 7:**
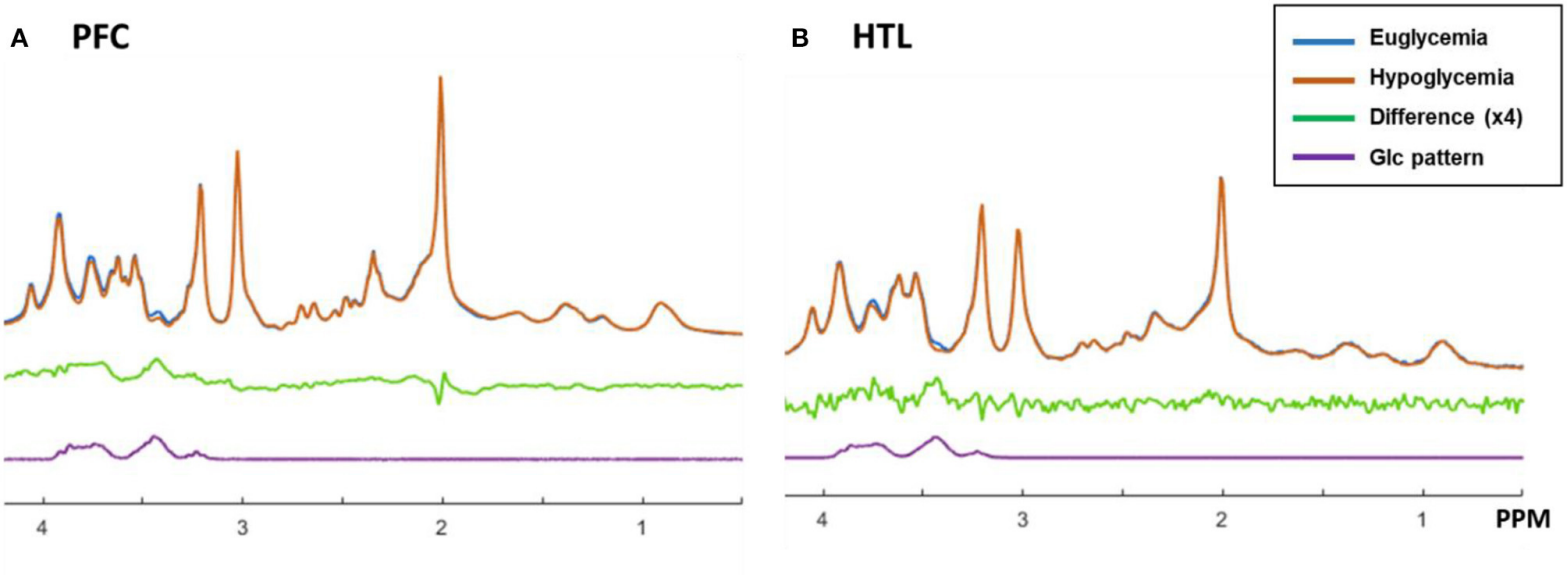
Proton MR spectra of prefrontal cortex (PFC, **A**) and hypothalamus (HTL, **B**) acquired during euglycemia (blue) and hypoglycemia (orange) and averaged across subjects (*N* = 6). The difference between spectra acquired during euglycemia and hypoglycemia is shown in green together with the spectral pattern of glucose in purple.

The spectral quality allowed reliable (mean CRLB ≤ 20%) estimation of the concentrations of 11 metabolites (GABA, Gln, Glu, GSH, Ins, Lac, PE, PCho+GPC, Cr+PCr, NAA+NAAG, and Glc+Tau) for the HTL and 12 metabolites (all HTL metabolites and Asp) for the PFC (Supplementary Table). Changes in Glc+Tau in response to glycemic changes were seen in both the PFC and HTL, which is attributable to changes in brain Glc (21). Visual inspection of between-subject averaged euglycemia and hypoglycemia spectra and their difference showed a lower Glc contribution in the hypoglycemia vs. euglycemia spectra (Figure 7).

**Figure 8:**
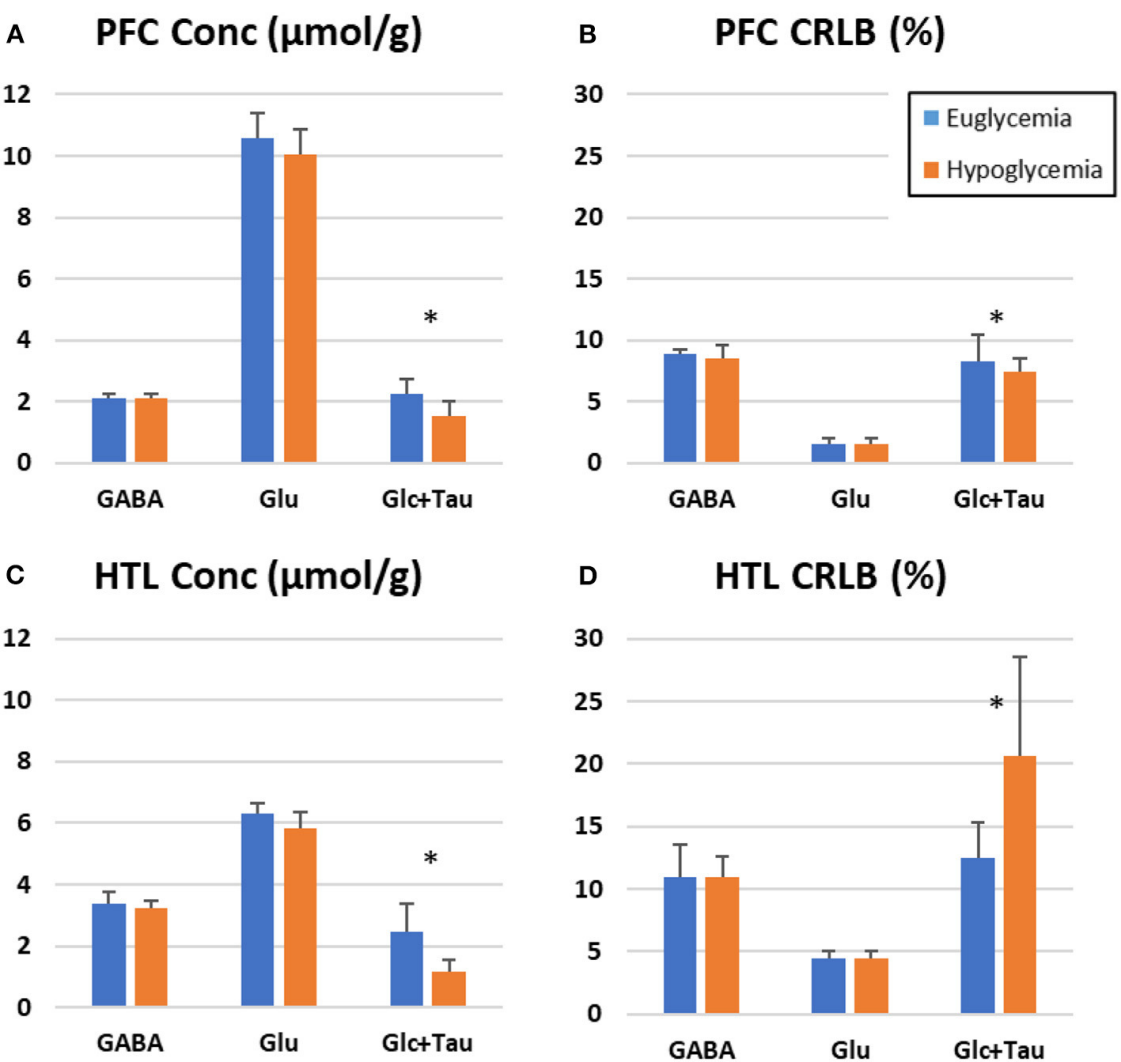
LC Model estimation of metabolites-of-interest for prefrontal cortex (PFC, **A,B**) and hypothalamus (HTL, **C,D**) during euglycemic and hypoglycemic glucose clamps-bar graph represents the mean value across participants (*N* = 6) with error bars representing the standard deviation. (Stars indicate *p* < 0.05, paired 2-tailed *t*-tests).

Metabolite level estimations confirmed lower level of Glc+Tau in hypoglycemia than euglycemia spectra for both the PFC (*p* = 0.02) and HTL (*p* = 0.02) (Supplementary Table). In addition, a trend for lower mean Glu values was observed during hypoglycemia vs. euglycemia in both the PFC and HTL (*p* = 0.1) (Figure 8).

## Discussion

### Overview

In this study, we developed and evaluated a highly optimized protocol for reliable acquisition of MRS data at UHF using commercial 7T hardware during lengthy clamp studies in the scanner where blood Glc is maintained at targeted ranges. The proposed protocol and in particular optical motion tracking offer highly improved data stability over our previous protocol for the HTL (11). Use of BaTiO_3_ dielectric padding enables data acquisition in the cortical regions of the brain using the widely available Nova coil.

### Dielectric Pad

We found that BaTiO_3_ padding of 5.5-mm thickness allowed sufficient RF power delivery to the PFC region (Figure 5). A thicker pad with 7-mm thickness did not fit inside the coil. Using simulations, we also found that 90° excitation required 26.1 μT and that 18.8 μT was the minimum threshold for adiabatic refocusing. RF power delivery below 18.8 μT is expected to yield sub-adiabatic refocusing in addition to insufficient excitation with the sLASER pulse sequence. The BaTiO_3_ pads with 5.5-mm thickness yielded sufficient RF power delivery for excitation (close to 26.1 μT) and adiabatic refocusing (>18.8 μT) to both the PFC and HTL with the 7T commercial single-transmit head coil. Comparison of the MRS data with vs. without padding showed SNR gains when using the BaTiO_3_ padding on the forehead, with PFC VOI showing large improvement (54–82%) in SNR over the dataset acquired without the pad.

The gain in SNR was relatively small for the HTL, and the padding on the forehead had minimal impact on the RF power transmitted to the HTL region. RF transmission to the HTL VOI is already sufficient with the single-channel Tx Nova coil without BaTiO_3_ padding because of the location of the VOI in the center of the brain with peak RF delivery. Note that in some participants, peak ${\text{B}}_{1}^{+}$ did not perfectly overlap our PFC or HTL VOI, as we had to avoid placing the BaTiO_3_ pad over the participant's eyes. Nonetheless, we were still able to observe a drastic improvement in the SNR over datasets collected without BaTiO_3_ pads.

### Evaluation of the Protocol During Glucose Clamp

Testing the protocol during long hypoglycemic Glc clamps on six patients showed excellent data quality with our proposed methodology. Motion tracking with dynamic shim updates allowed consistent data quality throughout the 22-min HTL acquisition, as well as between the euglycemia and hypoglycemia. This is critical especially for challenging VOI such as the HTL where almost half the datasets had to be excluded due to subject motion in a similar MRS study during a hypoglycemic clamp (11). The camera-based motion tracking allows high sensitivity to minor motion by detecting sub-millimeter displacements. For small VOIs such as the HTL (13 × 12 × 10 mm^3^), a displacement of few millimeters substantially displaces the VOI to unintented brain regions. The technical improvement alleviates the issue of subject motion and offers increased flexibility when the scan is accompanied with the complex Glc clamp procedures.

LCModel fitting of the processed spectra allowed the reliable quantification of an extended neurochemical profile in both the PFC and HTL regions. With the techniques to track and correct for motion, the proposed protocol allowed almost all of the transients collected on the HTL to be used for the analysis, which yielded an increase in SNR in the summed spectra and number of identifiable metabolites by LCModel. Thereby, we were able to reliably quantify 11 metabolites from this challenging brain region, a higher number than prior reports in human subjects (11, 42, 43). The newly quantifiable metabolites include GSH, Lac, and PE, which could provide additional information on neurochemical dynamics in response to changing glycemia. Importantly, the number of reliably quantified metabolites was similar between the two regions (12 vs. 11) despite PFC having much larger volume (8.64 mL) over the HTL (1.56 mL). Estimated concentrations (mM) and corresponding CRLB (%) showed a good reproducibility between datasets acquired at euglycemia and hypoglycemia.

With similar spectral quality between euglycemia and hypoglycemia, we were able to observe the effects of hypoglycemia on neurochemical concentrations. The expected Glc decline in the brain upon hypoglycemia is reliably detected by changes in the levels of Glc+Tau concentrations (Figure 8). We also observed a trend for lower mean Glu level with hypoglycemia in both VOI, consistent with prior observations in the OCC (10, 44). Other metabolites largely remained unchanged in response to hypoglycemia in this small sample, which reflects the brain's ability to maintain metabolic homeostasis despite drastic changes in Glc availability.

The hypoglycemic Glc clamp was designed to reproduce the real-life condition where the plasma Glc levels dropped from euglycemia to hypoglycemia. In theory, we could start the experiment with euglycemia outside the scanner, then drop blood Glc level, scan the participant, and finally increase the blood Glc back to euglycemia in the scanner for temporal randomization. However, this would be an even more challenging study design with increased scan time and resources to the current design, while adding little clinical value to our overall goal of studying the brain's response to acute hypoglycemia. Hence, such a design was not considered.

### Limitations

The sample size (*N* = 6) for the data acquired in patients with diabetes was small since these data were acquired to technically evaluate the protocol and not intended to statistically test hypotheses about cerebral responses to hypoglycemia. Hence, data with larger sample sizes will be needed to confirm whether the Glu trend observed in both VOI is significant.

In addition, even though our proposed protocol incorporated as much commercial solutions as possible, several of our components such as the BaTiO_3_ pad and nose attachment for the motion tracking marker had to be built in-house. A tooth attachment is now commercially available from KinetiCor for use in volume coils and will be incorporated in future studies. In addition, dielectric padding for neuroimaging is becoming commercially available with wider access to 7T scanners, including two FDA-approved 7T platforms.

Finally, while these experiments are challenging to implement, it is not possible to reduce the time and the complexity of these studies because of the large variation in insulin sensitivity seen among different people and the risks associated with hypoglycemia. The insulin infusion rate we administered was selected to cause hypoglycemia in humans with average insulin sensitivity, which may vary if the subject is either highly sensitive or resistant to insulin. Subjects who are very sensitive to insulin drop their blood sugar quite quickly in response to this infusion. As hypoglycemia is associated with a risk of unconsciousness, seizure, arrhythmia, and death, constant plasma Glc level monitoring along with adjustment of insulin infusion must be performed at all times. In addition, it takes about 20 min before the brain and plasma Glc levels come into equilibrium after a new target glycemia has been reached in the blood (45), meaning that sufficient time needs to pass before acquiring data in the brain at the target glycemia. Therefore, these challenging experimental procedures are intended for mechanistic studies at specialized research centers. Access to an UHF platform is necessary for optimal reproducibility of weakly represented metabolites and small VOI (37). On the other hand, the 3T would be the preferred platform if the primary metabolite of interest is Glc because Glc is quantified more reliably at 3T than 7T due to a simpler spectral pattern at the lower field (21, 37).

## Conclusion

Here, we demonstrate a highly optimized 7T MRS protocol that combined a commercial RF coil, BaTiO_3_ dielectric padding, optical motion tracking and frequency, and B_0_ shim corrections to ensure optimal stability of data quality during a challenging infusion protocol to maintain euglycemic and hypoglycemic blood Glc levels in clinical cohorts. The use of BaTiO_3_ padding boosted RF energy delivery to the PFC, allowing adiabaticity in sLASER at short TE and thereby optimal SNR and localization. Real-time motion, frequency, and B_0_ shim correction enabled acquisition of high-quality spectra throughout the prolonged MR sessions. The proposed protocol will allow robust mechanistic investigations of the primary neurotransmitters, Glu and GABA, under changing glycemic conditions.

## Data Availability Statement

The raw data supporting the conclusions of this article will be made available by the authors, without undue reservation.

## Ethics Statement

The studies involving human participants were reviewed and approved by University of Minnesota Institutional Review Board. The patients/participants provided their written informed consent to participate in this study.

## Author Contributions

GÖ and ES conceived and planned the experiments. DD and JJ performed technical implementation of the proposed pipeline. YP, DD, AK, AA, and ES carried out the data acquisition. YP and DD performed data analysis. YP, DD, AM, ES, and GÖ contributed to the interpretation of the results. YP wrote the manuscript. All authors provided critical feedback and helped shape the research, analysis, and manuscript.

## Conflict of Interest

The authors declare that the research was conducted in the absence of any commercial or financial relationships that could be construed as a potential conflict of interest.

## Publisher's Note

All claims expressed in this article are solely those of the authors and do not necessarily represent those of their affiliated organizations, or those of the publisher, the editors and the reviewers. Any product that may be evaluated in this article, or claim that may be made by its manufacturer, is not guaranteed or endorsed by the publisher.
