# Supplementary material for: Monitoring the Neurotransmitter Response to Glycemic Changes Using an Advanced Magnetic Resonance Spectroscopy Protocol at 7T
Source: Front Neurol. 2021 Aug 18;12:698675. doi: 10.3389/fneur.2021.698675 (PMC8416271; doi:10.3389/fneur.2021.698675)
Supplement: Supplementary file 1 [file Table_1.DOCX]

**Supplementary Table 1.** LCModel estimated concentrations on metabolites with mean CRLB ≤ 20%

| n = 6 | **Concentration, mM** | | | | | |
| --- | --- | --- | --- | --- | --- | --- |
| mean (SD) | **PFC** | | | **HTL** | | |
|  | **Euglycemia** | **Hypoglycemia** | **p-value** | **Euglycemia** | **Hypoglycemia** | **p-value** |
| **Asp** | 2.4 (0.3) | 2.6 (0.3) | 0.10 | - | - | - |
| **GABA** | 2.1 (0.1) | 2.1 (0.2) | 0.94 | 3.4 (0.3) | 3.2 (0.2) | 0.30 |
| **Gln** | 3.2 (0.7) | 3.2 (0.4) | 0.88 | 2.4 (0.3) | 2.3 (0.8) | 0.64 |
| **Glu** | 10.6 (0.9) | 10.0 (0.6) | 0.10 | 6.3 (0.3) | 5.8 (0.5) | 0.10 |
| **GSH** | 1.4 (0.1) | 1.4 (0.1) | 0.29 | 1.5 (0.2) | 1.4 (0.3) | 0.83 |
| **Ins** | 7.4 (0.8) | 7.4 (0.5) | 0.97 | 10.6 (1.3) | 10.3 (1.4) | 0.39 |
| **Lac** | 0.8 (0.2) | 0.7 (0.2) | 0.03 | 1.1 (0.1) | 1.1 (0.3) | 0.91 |
| **PE** | 3.6 (0.2) | 3.7 (0.2) | 0.37 | 2.3 (0.4) | 2.1 (0.6) | 0.14 |
| **PCho+GPC** | 2.0 (0.3) | 1.9 (0.2) | 0.46 | 3.0 (0.5) | 2.8 (0.4) | 0.20 |
| **Cr+PCr** | 7.9 (0.7) | 7.6 (0.3) | 0.29 | 7.7 (1.1) | 7.3 (0.6) | 0.38 |
| **NAA+NAAG** | 11.4 (0.6) | 10.9 (0.4) | 0.17 | 10.5 (1.1) | 9.8 (0.8) | 0.16 |
| **Glu+Gln** | 13.7 (1.3) | 13.2 (0.8) | 0.25 | 8.7 (0.2) | 8.1 (1.2) | 0.20 |
| **Glc+Tau** | 2.3 (0.5) | 1.5 (0.1) | 0.02 | 2.5 (0.9) | 1.2 (0.4) | 0.02 |

**Supplementary Table 2.** CRLB values from LCModel estimation of metabolites with mean CRLB ≤ 20%

| n = 6 | **CRLB, %** | | | | | |
| --- | --- | --- | --- | --- | --- | --- |
| mean (SD) | **PFC** | | | **HTL** | | |
|  | **Euglycemia** | **Hypoglycemia** | **p-value** | **Euglycemia** | **Hypoglycemia** | **p-value** |
| **Asp** | 13.2 (1.2) | 11.8 (1.0) | 0.12 | - | - | - |
| **GABA** | 8.8 (0.4) | 8.5 (1.0) | 0.47 | 11.0 (2.5) | 11.0 (1.5) | 1.00 |
| **Gln** | 5.0 (0.9) | 4.8 (0.8) | 0.36 | 12.7 (2.5) | 14.8 (8.8) | 0.57 |
| **Glu** | 1.5 (0.5) | 1.5 (0.5) | 1.00 | 4.5 (0.5) | 4.5 (0.5) | 1.00 |
| **GSH** | 5.3 (0.5) | 5.3 (0.8) | 1.00 | 11.0 (3.6) | 10.5 (2.2) | 0.81 |
| **Ins** | 2.0 (0.0) | 1.8 (0.4) | 0.36 | 2.0 (0.0) | 2.2 (0.4) | 0.36 |
| **Lac** | 13.0 (3.8) | 15.0 (4.8) | 0.08 | 18.2 (4.0) | 18.3 (3.3) | 0.95 |
| **PE** | 5.3 (0.5) | 5.0 (0.6) | 0.18 | 18.5 (4.6) | 20.2 (8.0) | 0.57 |
| **PCho+GPC** | 1.5 (0.5) | 1.7 (0.5) | 0.61 | 2.5 (0.5) | 2.3 (0.5) | 0.61 |
| **Cr+PCr** | 1.2 (0.4) | 1.0 (0.0) | 0.36 | 2.0 (0.0) | 2.0 (0.0) | 1.00 |
| **NAA+NAAG** | 1.0 (0.0) | 1.0 (0.0) | 1.00 | 2.0 (0.0) | 2.0 (0.0) | 1.00 |
| **Glu+Gln** | 1.8 (0.4) | 2.0 (0.0) | 0.36 | 4.2 (0.4) | 4.5 (0.5) | 0.18 |
| **Glc+Tau** | 8.3 (2.1) | 7.5 (1.0) | 0.22 | 12.5 (2.8) | 20.7 (7.9) | 0.09 |
